# Postpartum common mental disorders and its associated factors in eastern Ethiopia: a community-based cross-sectional study

**DOI:** 10.3389/fgwh.2025.1484834

**Published:** 2025-06-13

**Authors:** Dejene Tesfaye, Tadesse Misgana, Berhe Gebremichael, Daniel Alemu, Dawit Tamiru, Adisu Birhanu Weldesenbet, Mandaras Tariku, Merga Dheresa

**Affiliations:** ^1^ Department of Psychiatry, College of Health and Medical Sciences, Haramaya University, Harar, Ethiopia; ^2^ School of Public Health, College of Health and Medical Sciences, Haramaya University, Harar, Ethiopia; ^3^ Department of Midwifery, College of Health and Medical Sciences, Haramaya University, Harar, Ethiopia; ^4^ School of Nursing and Midwifery, College of Health and Medical Sciences, Haramaya University, Harar, Ethiopia

**Keywords:** common mental disorder, postpartum, social support, wealth index, intimate partner violence, community-based, associated factors, Eastern Ethiopia

## Abstract

**Introduction:**

Common mental disorders (CMD) cause marked emotional distress and interfere with daily function among postpartum women. In addition, the negative attitude towards mental disorders and their treatments prevents the women from seeking mental healthcare. Very little is known about CMD among women, especially in the postpartum period. This study, therefore, aimed at assessing the prevalence of CMD and its associated factors among women in the postpartum period at Kersa and Haramaya Health and Demographic Surveillance Sites (HDSS) sites, in eastern Ethiopia, between 1 July 2021 and 28 February 2022.

**Methods:**

This study employed a community-based cross-sectional study design using a quantitative method and was conducted in Kersa and Haramaya HDSS among 1,016 postpartum women. A structured questionnaire was used to collect data through face-to-face interviews about the variables related to sociodemographic and economic, clinical, psychosocial, substance use, and obstetric complication characteristics. The CMD was assessed by using the Self-Reporting Questionnaire (SRQ-20). A bivariable and multivariable logistic regression analysis was performed. All the variables with a *p*-value <0.25 in bivariable logistic regression were taken to multivariable logistic regression. Variables with a *p*-value < 0.05 in the multivariable regression were declared statistically significant associations. The odds ratio (OR) and 95% confidence intervals (CI) were used to show the strength of the association.

**Result:**

The prevalence of postpartum CMD was 23.84% (95% CI: 21.21–26.47). Among pregnant women who had CMD, only 103 (27.7%) had CMD and persisted to the postpartum period. Poor social support [adjusted OR (aOR): 1.88, 95% CI: 1.28–2.74], wealth index in the first quantile (aOR: 1.59, 95% CI: 1.06–2.39), presence of obstetric complication (aOR: 7.74, 95% CI: 4.38–13.69), and cesarean delivery (aOR: 5.01, 95% CI: 1.14–22.13) were the factors that showed a statistically significant association with postpartum CMD.

**Conclusion:**

One in every four study participants had CMD, which was higher than in studies conducted in Ethiopia among postpartum women. Social support, wealth index, obstetric complications, and mode of delivery were the factors with statistically significant associations. Postpartum women may benefit from the early diagnosis and treatment of CMD at the community and the primary healthcare level, and the integration of mental healthcare into maternal health services.

## Introduction

Common mental disorders (CMDs) include depression, mixed depression and anxiety, anxiety, and somatic symptoms. They cause marked emotional distress and interfere with daily function but do not usually affect insight or cognition (1–3).

During the postpartum period, severe mental illness can manifest as a continuation of a chronic psychotic condition that began during pregnancy or as an episode of severe mental illness that has a rapid onset soon after childbirth. The later episodes of postpartum or puerperal psychosis are usually characterized by mania, severe psychotic depression, or mixed episodes of high and low moods (4).

In a Canadian study, postpartum women who rated their physical health as high had approximately seven times greater odds of also reporting high mental health compared to those with lower self-rated physical health (5). Sociocultural adversity increases a woman’s risk of developing mental illness. For example, domestic violence during pregnancy negatively affects maternal wellbeing and often leads to poor engagement with healthcare services. In addition, the stigma surrounding mental illness may contribute to underreporting of mental health conditions (6).

A Vietnamese study revealed that 18.5% of women experienced CMDs within a year (7). In a South African study, maternal mental health was found to deteriorate over the postpartum period, with 49.1% of women reporting an increase in CMD symptoms from a few weeks after birth to several months later, compared to only 26.8% reporting a decrease (8).

A study conducted in South Africa reported a 16.4% prevalence of maternal distress (9). In Malawi, 13.9% of postpartum women were found to have current major depressive episodes, and 30.4% experienced some form of mental distress (10). In Ethiopia’s Amhara regional state, a cross-sectional survey of 1,319 women who had delivered within the previous 24 months revealed that 32.8% had probable CMD (11) based on a 4/5 cutoff score, compared to 19.8% using the more conservative 7/8 cutoff (3). Another Ethiopian study found a CMD prevalence of 18.6% among postpartum women (12), while a study conducted in Butajira reported a 5% prevalence during the first 2 months postpartum (13). In addition, a study presented by Gojjam Limenih at Parkwood Institute of Research on 27 April 2023 reported a CMD prevalence of 32% in Addis Ababa.

One South African study revealed that exposure to societal stressors and difficulty with a partner were reported to be significantly associated with maternal mental distress (9). Another South African study revealed that CMD was associated with unemployment (8). In the study from Malawi, low socioeconomic status, lack of confiding relationship with a partner or relative, and recent infant illness were significantly associated with maternal mental distress (10).

In the study conducted in the Amhara region, Ethiopia, unmarried women were more likely to experience poor mental health compared to their married counterparts (3). Another study conducted in same region showed that delivery at a health facility, having a sick/stillborn infant, and insufficient social support were significantly associated with postpartum CMD (14).

Despite the limited number of studies conducted in Ethiopia, existing research indicates a notably high prevalence of CMDs among women during pregnancy and the postpartum period. This study aimed to assess the prevalence of CMD and identify associated factors. The findings will provide clues about the magnitude of the issue and enhance further studies using more robust study designs to generate stronger evidence.

## Methods and materials

### Study setting, period, and study design

This study employed a community-based cross-sectional study design using quantitative methods. It was conducted between 1 July 2021 and 28 February 2022 in the Kersa and Haramaya Health and Demographic Surveillance Sites (HDSS) in eastern Ethiopia. Kersa HDSS is situated in Kersa District, Oromia Regional State, comprising 35 rural sub-districts (*kebeles*) and three small-town kebeles. According to the 2007 national census, the district had a total population of 172,626, with more than 6.9% residing in urban areas. The Kersa HDSS covers 24 of the 38 kebeles, and includes four health centers and 10 health posts. In 2013, the population in the Kersa HDSS was 60,694, with a male-to-female sex ratio of 1.02:1 (15). The Haramaya HDSS, established in 2018, encompasses 12 rural kebeles. At baseline, it included 17,461 households with a total population was 99,898 (51,259 boys/men and 48,639 girls/women), of whom 23.86% were women of reproductive age (16). During the data collection period, approximately 5,000 pregnant women were present in the study areas. The primary aim of the project was to assess the impact of maternal common mental disorders on obstetric outcomes, birth outcomes, infant nutritional status, and maternal functioning within the Kersa and Haramaya Health and Demographic Surveillance Sites. These sites were also used in other studies (17–20).

### Characteristics of the study population

This study is part of a large follow-up study titled “Impact of maternal common mental disorder on the obstetric outcome, birth outcomes, infant nutritional status and maternal functioning in Kersa and Haramaya HDSS, Eastern Ethiopia.” In this study, the source population included all women who gave birth within the Kersa and Haramaya HDDS sites. The study population comprised all women who delivered in the selected kebeles between 1 July 2021 and 28 February 2022. These women contributed data to various parameters of the main project (17–20). All the selected women were assessed for CMDs during the postpartum period (within 42 days of delivery).

### Data collection measurements

Structured and semi-structured questionnaires were used to collect data on participants' CMDs during the postpartum period, along with sociodemographic variables such as age, marital status, religion, ethnicity, educational status, occupation, and wealth index. Additional information was gathered on reproductive history, obstetric complications, gynecological characteristics, history of abortion, antenatal care follow-up, gynecological problems and operations, intimate partner violence (IPV), social support, and substance use. Substance use data included lifetime and current use of tobacco, khat, and alcohol. Validated and standardized instruments were used for data collection, including the Self-Reporting Questionnaire (SRQ-20), the WHO Multi-Country Study on Women’s Health and Domestic Violence Questionnaire, and the Oslo Social Support Scale (OSSS-3). Common mental disorders were assessed using the SRQ-20, which consists of 20 yes/no questions addressing symptoms of depression, anxiety, panic, and somatic complaints in the preceding 30 days. For this study, the SRQ-20 total score was dichotomized: scores <6 indicated the absence of CMDs, while scores ≥6 indicated the presence of CMDs (21). The SRQ-20 was translated into Amharic and validated in the Ethiopian general population. A cutoff point of 6 yielded a sensitivity of 90.7% and specificity of 80.7%. In the current study, the internal consistency was checked (Kappa value = 0.8).

Ever-use of substances was assessed using questions adapted from previously published literature (22, 23). Participants who responded “yes” to a question regarding lifetime use of a substance were classified as ever-users. Current substance use was similarly assessed based on literature-derived questions, with participants considered current users if they reported using the substance during their most recent pregnancy (22, 23). Household economic status was measured using a set of questions adapted and modified from the 2016 Ethiopian Demographic and Health Survey (EDHS) (24). IPV was evaluated using the WHO Multi-Country Study on Women’s Health and Domestic Violence questionnaire. This tool includes questions covering psychological, physical, and sexual violence, often accompanied by controlling behaviors. A single positive response to any of these questions indicated the presence of violence (25).

Social support was measured using the Oslo 3-item Social Support Scale (OSSS-3). The OSSS-3 includes three items that assess the number of close confidants, the perceived level of concern from others, and the perceived ease of obtaining help from neighbors. The total score was categorized into three levels: 3–8 indicating poor support; 9–11 moderate support; and 12–14 strong support (26). The OSSS-3 has previously been used in a population-level study in Ethiopia (27).

### Wealth index

To assess economic or wealth status, data were gathered on household properties and assets using a tool adapted from the 2016 Ethiopia Demographic and Health Survey (EDHS). Principal component analysis was then used to classify households into tertiles (three equal categories) based on their wealth index: 1st, 2nd, and 3rd tertiles (28).

### Sample techniques and procedures

To determine the minimum required sample size, we used a single population proportion formula with the following assumptions: a standard value (Z*_α_*_/2_) of 1.96 for a 95% confidence level, a 4% margin of error, a design effect (deff) of 1.5, and a proportion of CMD (*p*) among postpartum women of 32.8%, based on a previous study conducted in the Amhara region of Ethiopia (3). This calculation resulted in a sample size of 795. After accounting for a 10% non-response rate, the final sample size was 875.

To estimate the sample size for factors associated with postpartum CMD, we used the double population proportion formula via OpenEpi online software. This resulted in a minimum required sample size that was smaller than the 875 calculated using the single population proportion formula. Therefore, 875 was retained as the “minimum required sample size.” However, since this study was part of a larger follow-up project titled “Impact of maternal common mental disorder on the obstetric outcome, birth outcomes, infant nutritional status, and maternal functioning in Kersa and Haramaya Health and Demographic Surveillance Site, Eastern Ethiopia” a total sample size of 1,160 was used.

**Figure 1:**
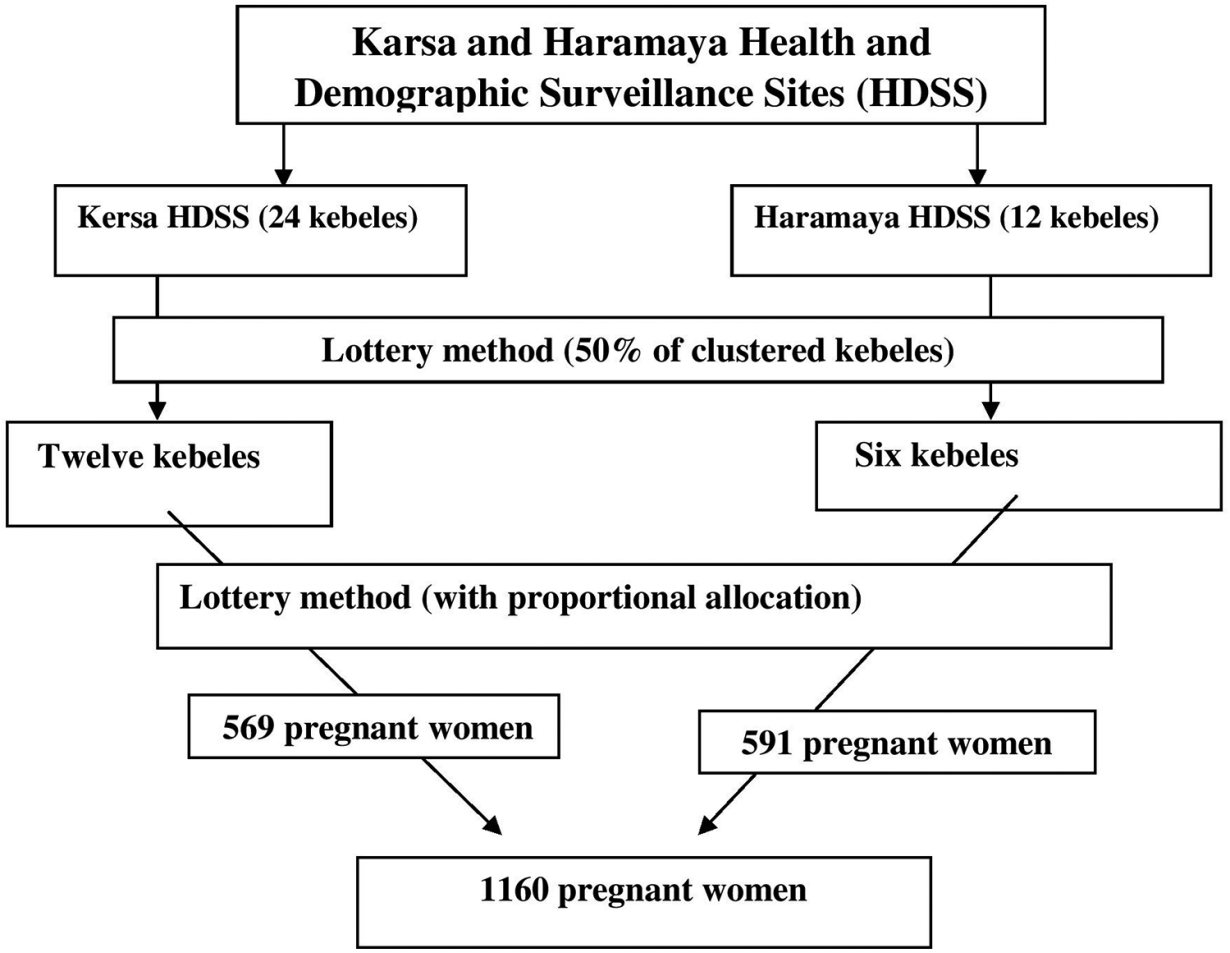
Schematic presentation of the sampling procedure for assessing postpartum common mental disorders and their associated factors among postpartum women.

There were 24 kebeles in Kersa and 12 kebeles in the Haramaya HDSS sites. From the total kebeles at each site, sample kebeles were selected using the lottery method. The total sample size was then proportionally allocated based on the number of pregnant women in the selected kebeles, and participants were also selected using the lottery method (Figure 1). This sampling procedure has been used in other studies conducted in the same setting (17–20). Approximately 140 of the pregnant women recruited had delivered before the start of data collection, and the study was finally conducted among 1,016 postpartum women.

### Data collection procedure

A total of 20 data collectors who have been working in Kersa and Haramaya Demographic Surveillance System (HDSS) sites collected the data for this study. In addition, six MSc holders supervised the data collection process. A 2-day training course was provided for the data collectors and supervisors. The data were collected via face-to-face interviews using structured and semi-structured questionnaires prepared in Open Data Kit collect forms, and completed data were sent directly to the server. The women were interviewed in separate rooms in their home environment or inside their compounds. The data collectors implemented WHO recommendations and precautions for the prevention of COVID-19 infection throughout the interviews. This data collection procedure has also been implemented by the same authors in other studies (17–20).

### Data quality assurance

The questionnaire was prepared in the English language and then translated into Afan Oromo and back into the English language by an expert in the language. Data collectors and supervisors received training. Before the actual data collection period, pre-testing was carried out on 10% of the sample size at Harar town. Supervisors and investigators closely supervised the data collectors during the data collection, a process that helped to maintain the consistency and completeness of the questionnaires.

### Statistical analysis

The data were collected, checked for completeness by a data clerk, cleaned, and coded. Data entry was done using EpiData (Version 3.1; EpiData Association, Denmark) and then exported to SPSS (Version 26; IBM Corp., Armonk, NY, USA) for analysis. Categorical variables were described as frequencies and percentages.

In the current study, both bivariable and multivariable logistic regression analyses were performed. Variables with at least one category showing a *p*-value of <0.25 in the bivariable analysis were included in the multivariable logistic regression model. The strength of association was expressed using odds ratios and 95% CI. A *p*-value <0.05 was considered statistically significant. Multicollinearity among independent variables was assessed using the variance inflation factor (VIF) and model fit was evaluated using the Hosmer–Lemeshow goodness-of-fit test.

## Results

### Sociodemographic and economic factors

The mean age of participants was 30.11 ± 8.48 years. Of the women, the majority (*n* = 673, 66.57%) were aged 20–35 years. Nearly all the participants were from the Oromo ethnic group (*n* = 1,006, 99.5%). Of the women, the majority (*n* = 769, 76.06%) were unable to read and write. Approximately 896 (88%) women were housewives. The majority (*n* = 978, 96.74%) of the women were married, and 388 (38.38%) of their households were in the first quantile of the wealth index (Table 1).

**Table 1:** Sociodemographic and economic characteristics of the postpartum women at Kersa and Haramaya HDSS sites, 2022.

S. No.	Variables	Category	Frequency	Percentage
	Age	<20 years	93	9.20
		20–35 years	673	66.57
		>35 years	245	24.23
	Ethnicity	Oromo	1,006	99.51
		Amhara	5	0.49
	Educational status	Literate	217	21.46
		Can read and write	25	2.47
		Neither read nor write	769	76.06
	Occupation	Farmer	47	4.65
		Housewife	896	88.63
		Others	68	6.73
	Marital status	Married	978	96.74
		Others	33	3.26
	Economic status	First tertiles	388	38.38
		Second tertiles	375	37.09
		Third tertiles	248	24.53

### Clinical, psychosocial, and substance use characteristics

This study showed that 25 (2.47%) study participants had a history of mental illnesses, and 23 (2.27%) had one or more medical illnesses. Nearly half (*n* = 491, 48.57%) of the women had experienced prenatal IPV. Approximately 440 (43.52%) participants had strong social support, and 242 (23.94%) had poor social support. One in five (20.47%) women were ever-users and one in every six (15.63%) were current users of any of the substances (tobacco, khat, alcohol, and others) (Table 2).

**Table 2:** Clinical, psychosocial, and substance use characteristics of the postpartum women at Kersa and Haramaya HDSS sites, 2022.

S. No.	Variables	Category	Frequency	Percentage
	Previous history of mental illness	Yes	25	2.47
		No	986	97.53
	Prenatal history of medical illness	Yes	23	2.27
		No	988	97.73
	Prenatal IPV	Yes	491	48.57
		No	520	51.43
	Social support	Strong	440	43.52
		Moderate	329	32.54
		Poor	242	23.94
	Ever use of substance	Yes	207	20.47
		No	804	79.53
	Ever tobacco	Yes	9	0.9
		No	1,002	99.1
	Ever Khat	Yes	204	20.2
		No	807	79.8
	Ever others	yes	7	0.7
		No	1,004	99.3
	Current use substance	Yes	158	15.63
		No	853	84.37
	Current tobacco	Yes	6	0.6
		No	1,005	99.4
	Current khat	Yes	157	15.5
		No	854	84.5
	Alcohol and other	Yes	4	0.3
		No	1,007	99.7

### Obstetric outcome

More than one-quarter (27.4%) of the women had at least one obstetric complication during pregnancy; headache and heartburn were the most common symptoms experienced by the women during the perinatal period. Approximately 80% (*n* = 810) of the women also reported that they had a planned pregnancy (Table 3).

**Table 3:** Obstetric complications and related characteristics of the postpartum women at Kersa and Haramaya HDSS sites, 2022.

S. No.	Variables	Categories	Frequency	Percentage
	Obstetric complication	Yes	277	27.40
		No	734	72.60
	Planned pregnancy	Yes	810	80.12
		No	201	19.88
	Perinatal nausea and vomiting	Yes	134	13.25
		No	877	86.75
	Perinatal headache	Yes	244	24.13
		No	767	75.87
	Heart burn	Yes	285	28.22
		No	725	71.78
	Edema	Yes	176	17.41
		No	835	82.59
	Back pain	Yes	181	17.90
		No	830	82.10
	Vaginal bleeding	Yes	99	9.79
		No	912	90.21
	Blurred vision	Yes	163	16.12
		No	848	83.88
	Eating non-nutritional substance	Yes	215	21.27
		No	796	78.73

### The prevalence of postpartum CMD

The prevalence of postpartum CMD was 23.84% (95% CI: 21.21–26.47). The mean SRQ-20 score among participants was 4.05 ± 4.01. There were differing rates of CMD symptoms. The most commonly reported symptoms were being easily frightened (39.37%), feeling easily tired (34.92%), and feeling tired all the time (26.61%). The least frequently reported symptoms were thoughts of ending one’s life (11.37%), feelings of worthlessness (13.16%), and difficulty thinking clearly (13.25%).

### Persistence and incidence of CMD

Among pregnant women who had higher levels of CMD during pregnancy, 103 (27.1%) continued to experience CMD in the postpartum period. In contrast, the incidence of postpartum CMD among women with low levels of CMD during pregnancy was 21.9% (*n* = 138).

### Factors associated with postpartum CMD

In total, 20 variables were tested using bivariable logistic regression. Of these, only 10 variables with a *p*-value of ≤0.25 were selected for inclusion in the multivariable logistic regression. These variables included age, social support, antenatal care follow-up, wealth index, prenatal CMD, prenatal IPV, planned pregnancy, presence of at least one obstetric complication, place of delivery, and mode of delivery. In the multivariable logistic regression analysis, four variables (social support, wealth index, presence of obstetric complication, and mode of delivery) showed a statistically significant association with postpartum CMD (*p* < 0.05). All four variables had a positive association with postpartum CMD (Table 4).

**Table 4:** Factors associated with CMD among the postpartum women at Kersa and Haramaya HDSS sites, 2022.

Explanatory variables	Categories	Postpartum CMD		OR (95% CI)	
		No	Yes	cOR (95%CI)	aOR (95%CI)
Age	<20 years	67	26	1.51 (0.87, 2.62)	1.60 (0.90, 2.87)
	20–35 years	508	165	1.27 (0.89, 1.81)	1.25 (0.85, 1.83)
	>35 years	195	50	1	1
Social support	Strong	358	82	1	1
	Moderate	251	78	1.36 (0.96, 1.92)	1.20 (0.83, 1.74)
	Poor	161	81	2.20 (1.53, 3.15)	1.88 (1.28, 2.74)***
ANC follow-up	Yes	361	127	1	1
	No	409	114	0.79 (0.59, 1.06)	0.77 (0.56, 1.05)
Wealth index	First tertiles	282	106	1.49 (1.02, 2.18)	1.59 (1.06, 2.39)**
	Second tertiles	290	85	1.16 (0.78, 1.72)	1.19 (0.79, 1.81)
	Third tertiles	198	50	1	1
Prenatal CMD	Yes	277	103	1.33 (0.99, 1.78)	1.03 (0.74, 1.43)
	No	493	138	1	1
Prenatal IPV	Yes	362	129	1.30 (0.97, 1.74)	1.28 (0.93, 1.77)
	No	408	112	1	1
Planned pregnancy	Yes	632	178	1	1
	No	138	63	1.62 (1.15, 2.28)	1.39 (0.97, 2.00)
Obstetric complication	Yes	14	227	4.81 (4.81, 14.72)	7.74 (4.38, 13.69)***
	No	263	507	1	1
Place of delivery	Health facility	301	111	1.33 (0.99, 1.78)	0.79 (0.57, 1.09)
	Home	469	130	1	1
Mode of delivery	SVD	754	228	1	1
	Instrumental	13	7	1.78 (0.70, 4.52)	1.14 (0.43, 3.08)
	Cesarean section	3	6	6.61 (1.64, 26.66)	5.01 (1.14, 22.13)*

Bold values indicate significantly associated factors.

ANC, antenatal care; CMD, common mental disorders; IPV, intimate partner violence; SVD, spontaneous vaginal delivery; cOR, crude odds ratio; aOR, adjusted odds ratio.

*p-value < 0.05.

**p-value < 0.01.

***p-value < 0.001.

## Discussion

This study assessed the prevalence of CMDs and their associated factors among women during pregnancy and the postpartum period (within 42 days after delivery). One in four (23.84%) women experienced CMD symptoms during the postpartum period (ongoing and new cases of CMD), while nearly one in seven (13.65%) women developed new CMD symptoms during the postpartum period.

The prevalence of CMD reported in this study is lower than findings from studies conducted in Malawi (10), the Amhara region (3), and Addis Ababa, Ethiopia. Several factors may explain this discrepancy. The Malawian study had a smaller sample size and included both major and minor mental disorders under CMD, which may have inflated the results. Similarly, the study from the Amhara region used a lower cutoff score for CMD compared to the current study, which could also lead to a higher reported prevalence. However, the findings of this study highlight th eneed for effective screening and intervention strategies to address CMD among postpartum women (29).

On the other hand, the prevalence of CMD in this study was higher than that reported in studies conducted in South Africa (9), the Gurage Zone of Ethiopia (12), and significantly higher than studies from Butajira (13, 30).

The discrepancy with the South African study was that it focused on postpartum depression alone and its participants were exclusively from an urban setting, whereas the majority of participants in our study were from rural settings. The Gurage Zone study had a much smaller sample size and included only women aged 18–37 years. The discrepancy with the Butajira study could be explained by the difference in the timing of data collection; our study assessed CMD in the early weeks of the postpartum period, whereas the Butajira study collected data 2 months after childbirth.

This study revealed that a significant number of women in low- and middle-income countries experience CMD, such as depression and anxiety, during the postpartum period (7). Other studies have shown that maternal CMD is a predictor for poverty, relationship difficulties, developmental issues in children (13, 14, 31).

A statistically significant positive association was found between postpartum CMD and a lack of social support. This evidence is supported by several population-based studies (32–34). For example, a study conducted in Malawi found that women without a confiding relationship – whether with a partner or relatives – were significantly more likely to experience postpartum CMD. Research from the Amhara region of Ethiopia also reported that inadequate social support was associated with increased odds of postpartum CMD (14). Social support is one of the most important factors that predicts maternal mental health during the postpartum period, with those lacking sufficient support more likely to experience stress, depression, and other mental disorders (35, 36).

This study also found a statistically significant positive association between postpartum CMD and the mother’s economic status, as shown in population-based studies (32–34) and a study conducted in Pakistan (37). The study from Malawi supported the association between low socioeconomic status and increased risk of postpartum CMD (10).

This study also revealed that mothers who experienced obstetric complications were more likely to experience postpartum CMD compared to those without such complications. Finally, the study revealed a significant association between caesarean delivery and postpartum CMD. This finding was also supported by a previous study (38).

This study used locally validated and standardized instruments in Ethiopia and was conducted as a community-based study, which can be considered a key strength. However, the use of a cross-sectional design limits the ability to establish cause-and-effect relationships between the independent and dependent variables.

CMD symptoms are associated with functional impairment in postpartum women (39). In addition, the presence of CMD in this population may be linked to an increased risk of suicide, indicating a significant mortality rate in this group of women (40). Therefore, these findings underscore the importance of early identification and intervention in addressing the issue of mortality. Given that postpartum women with mental health problems are less likely to seek mental health services, mental health interventions should prioritize these at-risk groups (41).

## Conclusion and recommendations

The prevalence of CMD among postpartum women was high, with a significant proportion experiencing persistent symptoms during the postpartum period. Social support, wealth index, obstetric complications, and mode of delivery were found to have a statistically significant association with postpartum CMD. Proactive measures during pregnancy and after childbirth may help reduce the occurrence of CMD. These include promoting a healthy lifestyle and addressing socioeconomic challenges. Integrating mental health services into primary healthcare and maternal health programs could alleviate the burden of CMD among postpartum women. Strengthening social support systems and improving the economic conditions of pregnant and postpartum women may also prevent mental health problems. Early detection and treatment of obstetric complications could reduce the risk of mental health issues. For women undergoing cesarean sections, comprehensive care and support would benefit them. Early identification and management of both physical and mental health conditions, alongside access to mental health services, are also essential.

## Data Availability

The raw data supporting the conclusions of this article will be made available by the authors, without undue reservation.

## References

[1] Stansfeld S Clark C Bebbington PE King M Jenkins R Hinchliffe S. Common mental disorders. In: McManus S Bebbington PE Jenkins R Brugha T, editors. *Mental Health and Wellbeing in England: Adult Psychiatric Morbidity Survey 2014*. Leeds: NHS Digital (2016). p. 37–68.

[2] Henderson M Harvey SB Øverland S Mykletun A Hotopf M. Work and common psychiatric disorders. J R Soc Med. (2011) 104(5):198–207. 10.1258/jrsm.2011.100231 21558098 PMC3089873

[3] Baumgartner JN Parcesepe A Mekuria YG Abitew DB Gebeyehu W Okello F Maternal mental health in Amhara region, Ethiopia: a cross-sectional survey. Glob Health Sci Pract. (2014) 2(4):482–6. 10.9745/GHSP-D-14-00119 25611481 PMC4307863

[4] Jones I Chandra PS Dazzan P Howard LM. Bipolar disorder, affective psychosis, and schizophrenia in pregnancy and the post-partum period. Lancet. (2014) 384(9956):1789–99. 10.1016/S0140-6736(14)61278-2 25455249

[5] Varin M Palladino E Orpana HM Wong SL Gheorghe M Lary T Prevalence of positive mental health and associated factors among postpartum women in Canada: findings from a national cross-sectional survey. Matern Child Health J. (2020) 24:759–67. 10.1007/s10995-020-02920-8 32323116 PMC7198477

[6] Smith MS Lawrence V Sadler E Easter A. Barriers to accessing mental health services for women with perinatal mental illness: systematic review and meta-synthesis of qualitative studies in the UK. BMJ Open. (2019) 9(1):e024803. 10.1136/bmjopen-2018-024803 30679296 PMC6347898

[7] Nguyen TT Tran TD Tran T La B Nguyen H Fisher J. Postpartum change in common mental disorders among rural Vietnamese women: incidence, recovery and risk and protective factors. Br J Psychiatry. (2015) 206(2):110–5. 10.1192/bjp.bp.114.149138 25395687

[8] Silverman DT Killion JP Evans D Coetzee L Rockers PC Hamer DH. Postpartum mental health in rural South Africa: socioeconomic stressors and worsening mental health. Matern Child Health J. (2022) 26:434–40. 10.1007/s10995-021-03268-3 34665355

[9] Ramchandani PG Richter LM Stein A Norris SA. Predictors of postnatal depression in an urban South African cohort. J Affect Disord. (2009) 113(3):279–84. 10.1016/j.jad.2008.05.007 18571734

[10] Stewart RC Bunn J Vokhiwa M Umar E Kauye F Fitzgerald M Common mental disorder and associated factors amongst women with young infants in rural Malawi. Soc Psychiatry Psychiatr Epidemiol. (2010) 45:551–9. 10.1007/s00127-009-0094-5 19609476

[11] Harpham T Reichenheim M Oser R Thomas E Hamid N Jaswal S Measuring mental health in a cost-effective manner. Health Policy Plan. (2003) 18(3):344–9. 10.1093/heapol/czg041 12917276

[12] Monaghan S Akale MA Demeke B Darmstadt GL. Prevalence and stigma of postpartum common mental disorders in the Gurage region of Ethiopia: a mixed-methods observational cohort study. Front Psychol. (2021) 12:626797. 10.3389/fpsyg.2021.626797 33897534 PMC8062741

[13] Medhin G Hanlon C Dewey M Alem A Tesfaye F Lakew Z The effect of maternal common mental disorders on infant undernutrition in Butajira, Ethiopia: the P-MaMiE study. BMC psychiatry. (2010) 10(1):1–13. 10.1186/1471-244X-10-32 20433695 PMC2879242

[14] Baumgartner JN Parcesepe A Mekuria YG Abitew DB Gebeyehu W Okello F Correlates of postpartum common mental disorders: results from a population-based study in Amhara region, Ethiopia. Arch Women’s Mental Health. (2016) 19:937–42. 10.1007/s00737-016-0617-5 26961004

[15] Assefa N Oljira L Baraki N Demena M Zelalem D Ashenafi W HDSS profile: the Kersa health and demographic surveillance system. Int J Epidemiol. (2016) 45(1):94–101. 10.1093/ije/dyv284 26510420 PMC4795560

[16] Girma Gudata Z Dheresa M Mengesha G Roba KT Yusuf J Daraje G Cohort profile: the Haramaya health and demographic surveillance system (Haramaya HDSS). Int J Epidemiol. (2021) 51:46–54. 10.1093/ije/dyab232 34738113

[17] Misgana T Tesfaye D Alemu D Gebremichael B Tamiru D Tariku M Khat use and associated factors during pregnancy in eastern Ethiopia: a community-based cross-sectional study. Front Glob Women’s Health. (2022) 3:941300. 10.3389/fgwh.2022.941300 36532956 PMC9757490

[18] Tamiru D Misgana T Tariku M Tesfaye D Alemu D Weldesenbet AB Prevalence and associated factors of common mental disorders among pregnant mothers in Rural Eastern Ethiopia. Front Psychiatry. (2022) 13:843984. 10.3389/fpsyt.2022.843984 35418883 PMC8995426

[19] Misgana T Weldesenbet AB Tamiru D Tariku M Tesfaye D Alemu D Intimate partner violence and its predictors among pregnant women in eastern Ethiopia: generalized structural equation modeling. Int J Reprod Med. (2022) 2022:7827234. 10.1155/2022/7827234 36035446 PMC9410972

[20] Gebremichael B Misgana T Tamiru D Tariku M Tesfaye D Alemu D Undernutrition and associated factors among rural pregnant women in eastern Ethiopia. SAGE Open Med. (2022) 10:20503121221104430. 10.1177/20503121221104430 35722439 PMC9201300

[21] Beusenberg M Orley JH, World Health Organization. A User’s Guide to the Self Reporting Questionnaire (SRQ). (No. WHO/MNH/PSF/94.8). Geneva: World Health Organization (1994).

[22] Mekuriaw B Belayneh Z Yitayih Y. Magnitude of khat use and associated factors among women attending antenatal care in Gedeo zone health centers, southern Ethiopia: a facility based cross sectional study. BMC Public Health. (2020) 20(1):1–8. 10.1186/s12889-019-8026-0 31992259 PMC6988234

[23] Alem A Kebede D Kullgren G. The prevalence and socio-demographic correlates of khat chewing in Butajira, Ethiopia. Acta Psychiatr Scand. (1999) 100:84–91. 10.1111/j.1600-0447.1999.tb10699.x 10470360

[24] Central Statistical Agency/CSA/Ethiopia and ICF. Ethiopia Demographic and Health Survey 2016. Addis Ababa, Ethiopia and Rockville, MD, USA: CSA and ICF (2016).

[25] García-Moreno C Jansen H Ellsberg M Heise L Watts C. WHO Multi-Country Study on Women’s Health and Domestic Violence Against Women: Initial Results on Prevalence, Health Outcomes and Women’s Responses. Switzerland: World Health Organization (2005).

[26] Kocalevent RD Berg L Beutel ME Hinz A Zenger M Harter M Social support in the general population: standardization of the Oslo social support scale (OSSS-3). BMC Psychol. (2018) 6(1):31. 10.1186/s40359-018-0249-9 30016997 PMC6050647

[27] Fekadu A Medhin G Selamu M Hailemariam M Alem A Giorgis TW Population level mental distress in rural Ethiopia. BMC Psychiatry. (2014) 14:194. 10.1186/1471-244X-14-194 24999041 PMC4227077

[28] Mengesha HG Vatanparast H Feng C Petrucka P. Modeling the predictors of stunting in Ethiopia: analysis of 2016 Ethiopian demographic health survey data (EDHS). BMC Nutr. (2020) 6:1–11. 10.1186/s40795-020-00378-z 32974038 PMC7507682

[29] MacLean JV Faisal-Cury A Chan Y-F Menezes PR Winters A Joseph R The relationship between sleep disturbance in pregnancy and persistent common mental disorder in the perinatal period (sleep disturbance and persistent CMD). J Mental Health. (2015) 24(6):375–8. 10.3109/09638237.2015.1036969 26382909

[30] Hanlon C Medhin G Alem A Araya M Abdulahi A Tomlinson M Sociocultural practices in Ethiopia: association with onset and persistence of postnatal common mental disorders. Br J Psychiatry. (2010) 197(6):468–75. 10.1192/bjp.bp.109.076497 21119153 PMC2994937

[31] Hoffman C Dunn DM Njoroge WF. Impact of postpartum mental illness upon infant development. Curr Psychiatry Rep. (2017) 19:1–6. 10.1007/s11920-017-0857-8 29105008

[32] Anselmi L Barros FC Minten GC Gigante DP Horta BL Victora CG. Prevalence and early determinants of common mental disorders in the 1982 birth cohort, Pelotas, southern Brazil. Rev Saude Publica. (2008) 42:26–33. 10.1590/S0034-89102008000900005 19142342 PMC2667238

[33] Patel V Kirkwood BR Pednekar S Weiss H Mabey D. Risk factors for common mental disorders in women: population-based longitudinal study. Br J Psychiatry. (2006) 189(6):547–55. 10.1192/bjp.bp.106.022558 17139040

[34] Rognmo K Torvik FA Røysamb E Tambs K. Alcohol use and spousal mental distress in a population sample: the Nord-Trøndelag health study. BMC Public Health. (2013) 13(1):1–13. 10.1186/1471-2458-13-319 23570535 PMC3649917

[35] Cho H Lee K Choi E Cho HN Park B Suh M Association between social support and postpartum depression. Sci Rep. (2022) 12(1):3128. 10.1038/s41598-022-07248-7 35210553 PMC8873474

[36] Yağmur Y Ulukoca N. Social support and postpartum depression in low-socioeconomic level postpartum women in eastern Turkey. Int J Public Health. (2010) 55:543–9. 10.1007/s00038-010-0182-z 20725761

[37] Rahman A Creed F. Outcome of prenatal depression and risk factors associated with persistence in the first postnatal year: prospective study from Rawalpindi, Pakistan. J Affect Disord. (2007) 100(1–3):115–21. 10.1016/j.jad.2006.10.004 17098291 PMC1894757

[38] Fatoye FO Adeyemi AB Oladimeji BY. Emotional distress and its correlates among Nigerian women in late pregnancy. J Obstet Gynaecol. (2004) 24(5):504–9. 10.1080/01443610410001722518 15369927

[39] Senturk V Hanlon C Medhin G Dewey M Araya M Alem A Impact of perinatal somatic and common mental disorder symptoms on functioning in Ethiopian women: the P-MaMiE population-based cohort study. J Affect Disord. (2012) 136(3):340–9. 10.1016/j.jad.2011.11.028 22196052 PMC3314986

[40] Wisner KL Sit DK McShea MC Rizzo DM Zoretich RA Hughes CL Onset timing, thoughts of self-harm, and diagnoses in postpartum women with screen-positive depression findings. JAMA Psychiatry. (2013) 70(5):490–8. 10.1001/jamapsychiatry.2013.87 23487258 PMC4440326

[41] Dagher RK Pérez-Stable EJ James RS. Socioeconomic and racial/ethnic disparities in postpartum consultation for mental health concerns among US mothers. Arch Women’s Mental Health. (2021) 24:781–91. 10.1007/s00737-021-01132-5 33855652

